# Ultrastructure and Glycoconjugate Pattern of the Foot Epithelium of the Abalone *Haliotis tuberculata* (Linnaeus, 1758) (Gastropoda, Haliotidae)

**DOI:** 10.1100/2012/960159

**Published:** 2012-05-03

**Authors:** I. Bravo Portela, V. S. Martinez-Zorzano, I. Molist- Perez, P. Molist García

**Affiliations:** ^1^Instituto Español de Oceanografía, Centro Oceanográfico de Vigo, Apdo. 1552, 36200 Vigo, Spain; ^2^Department of Biochemistry, Genetics and Immunology, University of Vigo, 36310 Vigo, Spain; ^3^Department of Functional Biology and Health Science, University of Vigo, 36310 Vigo, Spain

## Abstract

The foot epithelium of the gastropod *Haliotis tuberculata* is studied by light and electron microscopy in order to contribute to the understanding of the anatomy and functional morphology of the mollusks integument. Study of the external surface by scanning electron microscopy reveals that the side foot epithelium is characterized by a microvillus border with a very scant presence of small ciliary tufts, but the sole foot epithelium bears a dense field of long cilia. Ultrastructural examination by transmission electron microscopy of the side epithelial cells shows deeply pigmented cells with high electron-dense granular content which are not observed in the epithelial sole cells. Along the pedal epithelium, seven types of secretory cells are present; furthermore, two types of subepithelial glands are located just in the sole foot. The presence and composition of glycoconjugates in the secretory cells and subepithelial glands are analyzed by conventional and lectin histochemistry. Subepithelial glands contain mainly N-glycoproteins rich in fucose and mannose whereas secretory cells present mostly acidic sulphated glycoconjugates such as glycosaminoglycans and mucins, which are rich in galactose, N-acetyl-galactosamine, and N-acetyl-glucosamine. No sialic acid is present in the foot epithelium.

## 1. Introduction

The epithelium of mollusks usually consists of a prismatic single layer of three main cell types: microvillous, ciliated, and glandular cells. This general common structure is diversified and enriched by different cell types depending on the specialization of the epithelia to particular functions. The variations in structure and functions of mollusk integument have been well summarized by Bubel [1] and Simkiss [2] who describe how different functions correspond to different cell types, diverse secretions of the glandular cells, and the existence of ciliated or microvilli-bearing epithelial cells.

Studies on structure and function of gastropod epithelia have been performed regarding the epithelium of specialized organs such as mantle [3–5], tentacles [6], and defensive glands [7]. Regarding the gastropod foot some research has focused on histological studies in muscular [8–11] and connective tissues [12]. However, although the epithelium of the foot has received much more attention [13–18], histochemical and ultrarstructural studies on the vestigatropod pedal epithelium are scarce [19, 20].

Typically the gastropod foot is covered by a mucus layer important to a range of functions including lubrication, locomotion, protection, and adhesion to the substrate [21]. It has been showed the viscoelastic properties of limpet mucus can be modified in different ways when a specific function is required [22, 23]. Moreover, the mucus layer has an important ecological role in the community behavior [24] and in the ecosystem, as defense or attraction. It also provides a habitat for microorganisms as it has been described in *Haliotis diversicolor* [25].

The mucus layer covering the gastropods foot is produced by epithelial secretory cells and subepithelial glands. The diversity of these cell types, their composition, and distribution vary from species to species. For instance Grenon and Walker [14] distinguished nine types of secretory cells in the foot epithelium of the marine gastropod, *Patella vulgata*, but a different distribution of secretory cells has been found by the same authors in another limpet *Acmaea tessulata*. However Shirbhate and Cook [18] described ten secretory cell types in another marine gastropod *Littorina littorea*. The conventional histochemical methods used in these studies revealed different types of secretory cells containing acidic and neutral glycoconjugates, but they yield incomplete information on the structural details of glycans. The presence of glycosaminoglycans and glycoprotein has been demonstrated, by using lectins, in the epithelium of a few mollusks [26, 27], but no data exist in the literature on the nature and distribution of glycoconjugates in the foot of the *Haliotis* species.

In addition to the secretory system, gastropods have been described as containing a variety of pigments in their epithelial cells, including carotenoid, melanin, and bilichromes among others [28]. In a previous study by using light microscopy, Bravo et al. [29] have characterized two types of pigmented cells located in the crests and grooves of the unfolded side foot of *Haliotis tuberculata *(Linnaeus, 1758). These cells contained respectively melanin and phycobiliprotein-like pigment, which give this integument its appearance and characteristic color.

The aim of this work is to characterize ultrastructurally the different cell types found in both side and sole foot epithelia of *Haliotis tuberculata*. In addition we analyze by using conventional and lectin histochemistry methods the distribution and composition of glycoconjugates in this epithelium in order to contribute to the understanding of the anatomy and functional morphology of the mollusks integument.

## 2. Material and Methods

### 2.1. Animals

Seven individual adults of *Haliotis tuberculata *about 12 cm in length were collected in different seasons along 2009 from different locations in the Ría of Vigo, (NW Spain). The habitat of *Haliotis* is infralitoral so a diver collected them from underwater rocks taking care not to damage the animals, and they were placed in seawater with aeration until they were processed into few hours. After they were anesthetized by immersion in 5% MgCl_2_ in seawater [30], small pieces of the foot were cut from the medium edge part of the animal body taken at the same time the lateral and ventral part of the foot as it is showed in our previous paper [29]. We analyzed in the same section the lateral part of the foot integument named side foot and the sole foot which is the surface of locomotion in contact with the substrate. All procedures for animal experimentation were approved by the animal care and use committee of the Regional Government of Galicia (Xunta de Galicia) and conformed to the guidelines of the European Community.

### 2.2. Light Microscopy

Samples were fixed in formol Baker (Prolabo) for 24–48 h at room temperature, washed in tap water, and embedded in paraffin. Sections (8 *μ*m thick) were deparaffinized in xylene, rehydrated with graded ethanol, and subjected to the following histochemical procedures for the identification of glycoconjugates.

#### 2.2.1. Conventional Histochemical Techniques

Sections were stained with alcian blue (AB, pH 1 or 2.5, Sigma) to demonstrate acidic glycoconjugates, and high iron diamine (HID) combined with alcian blue (HID/AB) for separating sulphated and carboxylated glycoconjugates. As a control some sections were desulphated before stained with AB or HID. Desulphation is a sequential process of methylation and saponification specifically applied to remove sulphate-ester groups [31].

Samples were also subjected to chemical method using periodic acid-Schiff reactive (PAS, Merck) which is positive for glycoconjugates containing neutral sugars and/or sialic acid. In histochemistry, the presence of neutral monosaccharide residues means that they do not have sulphate-ester, carboxylic acid, or nitrogen-containing functional groups. In order to know if the PAS-positive compounds were glycogen, an amylase test was carried out by using rat liver sections as a positive control. All staining protocols were performed according to Kiernan [31] and Molist et al. [32].

#### 2.2.2. Lectin Histochemistry

Lectins labeled with digoxigenin (DIG), from Boehringer Mannheim Biochemica, and biotin (Sigma) were used to identify specific sugar residues in glycoconjugates. Lectins conjugated with DIG were used following the method previously described by Fernández-Rodríguez et al. [33], but slightly modified. Briefly, sections were preincubated overnight in a moist chamber with blocking solution and rinsed twice in Tris buffer saline (TBS). Then, sections were incubated for 2 h at room temperature with the following DIG conjugated lectins: (*Galanthus nivalis*) GNA specific for mannose, (*Sambucus nigra*) SNA and *(Maackia amurensis) *MAA both specific for sialic acid, PNA (*Arachis hypogaea*) which recognizes the sequence Galactose (beta 1–3) N-acetyl-galactosamine, or (*Aleuria aurantia*) AAA specific for fucose, diluted in TBS containing different salts (1 mM MgCl_2_, 1 mM MnCl_2_, and 1 mM CaCl_2_). Afterwards, sections were rinsed three times in TBS, and incubated during 2 h with anti-digoxigenin-alkaline phosphatase (anti-DIG-AP) diluted 1 : 1000 with TBS. Finally, after three washes with TBS the AP activity was visualized with 5-bromo-4-cloro-3-indolylphosphate (BCIP)/4-nitroblue tetrazolium chloride (NBT), both from Boehringer Mannheim Biochemica. The reaction was stopped with distilled water, and sections were subsequently dehydrated, coverslipped, and analyzed with a BX51 Olympus microscope.

Lectins conjugated with biotin were used as the following: After inactivation of endogenous peroxidase activity with 3% of H_2_O_2_ in methanol for 30 min, sections were washed in TBS and preincubated with 1% bovine serum albumin in TBS to minimize nonspecific binding. Subsequently, sections were incubated overnight at 4°C with the following lectins: WGA (*Triticum vulgare*) specific for N-acetyl-glucosamine, DBA (*Dolichos biflorus*) specific for N-acetyl-galactosamine, UEA I (*Ulex europaeus*) and LTA (*Lotus tetragonolobus*) both specific for fucose, or ConA (*Canavalia ensiformis*) specific for mannose. Then, sections were rinsed in TBS and incubated for 1 h with the avidin biotin peroxidase reagent (ABC Kit Vector Laboratories) diluted 1 : 100 in the same buffer. This complex was developed with 3,3′diaminobenzidine tetrahydrochloride (DAB, tablets 0,7 mg/mL, Sigma) and 0,03% H_2_O_2_ in TBS for 5–10 min. Finally sections were rinsed with TBS, dehydrated, and mounted. Some sections were counterstained with Mayer's haematoxylin (Montplet & Esteban SA).

All lectins were employed at a concentration of 10 *μ*g/mL except for MAA and SNA, which were applied at three different concentrations (10, 25, and 50 *μ*g/mL). To verify the specificity of the lectins, two types of controls were performed: A general control where the conjugated lectin was substituted by buffer and a specific control by preincubation during 1 h of each lectin with its specific mono/oligosaccharide (Sigma) at the appropriate concentrations. In any case, no labeling was detected in the control sections.

Moreover, desulphation treatment was used before incubation with the lectins.

### 2.3. Scanning Electron Microscopy (SEM)

Pieces approximately 3-4 mm^3^ were fixed for 4 h in 2.5% glutaraldehyde (Merck) in filtered sea water. Samples were subsequently washed in cacodylate buffer, dehydrated in a graded series of ethanol, and isoamyl acetate (10 minutes each at room temperature), critical point dried in CO_2_, coated with gold, and examined with a Philips XL30 SEM.

### 2.4. Transmission Electron Microscopy (TEM)

Small pieces of the integument were fixed in 2% paraformaldehyde (Scharlau) and 2% glutaraldehyde in cacodylate buffer for approximately 3 h at 4°C. Then, tissues were washed three times (1 h each) in the same buffer and postfixed in 1% osmium tetroxide in cacodylate buffer for 2 h at 4°C. After rinsing the tissues in buffer, they were then dehydrated in a graded series of acetone and subsequently, embedded in Spurr's resin. Toluidine blue-(Sigma) stained semithin sections were used to determine the area of study. Ultrathin sections were stained with uranyl acetate and lead citrate and analyzed with either a Philips CM20 or a Jeol JEM1010 TEM.

## 3. Results

### 3.1. General Features and Scanning Electron Microscopy

**Figure 1:**
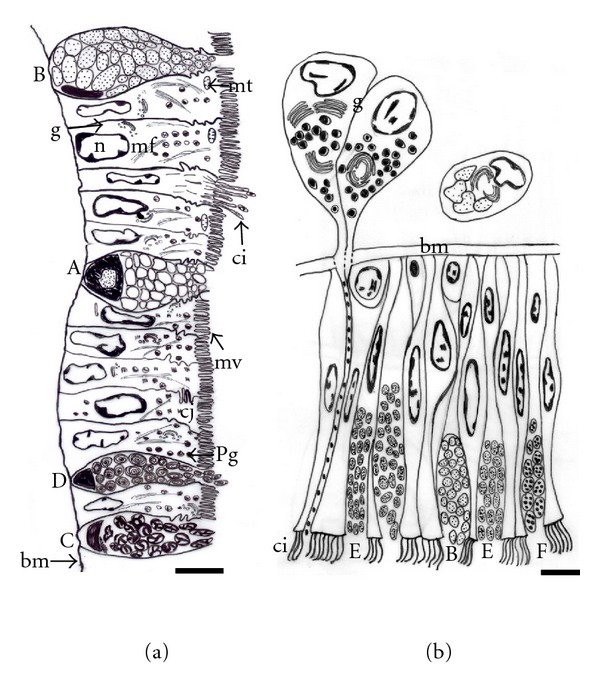
Schematic drawing of the side (a) and sole (b) foot epithelia. Secretory cells (A, B, C, D, E, and F), basal membrane (bm), cilia (ci), Golgi complex (g), cell junctions (cj), microfilaments (mf), mitochondria (mt), microvillus border (mv), nuclei (n), and pigmented granules (pg). Bar: 10 *μ*m (a), 5 *μ*m (b).

The foot epithelium ultrastructure of *Haliotis tuberculata* is schematized in the drawings shown in Figures 1(a) and 1(b). The side foot is lined by two types of columnar epithelial cells showing a prominent brush border interspersed with ciliated cells and four different types of epithelial secretory cells (Figure 1(a)). By contrast, the sole foot is characterized by taller columnar ciliated cells with three kind of epithelial secretory cells among them. Moreover clusters of secretory cells are embedded in the subepithelial space of the sole foot, and, due to their arrangement in glandular complexes, we refer to them as subepithelial glands (Figure 1(b)).

**Figure 2:**
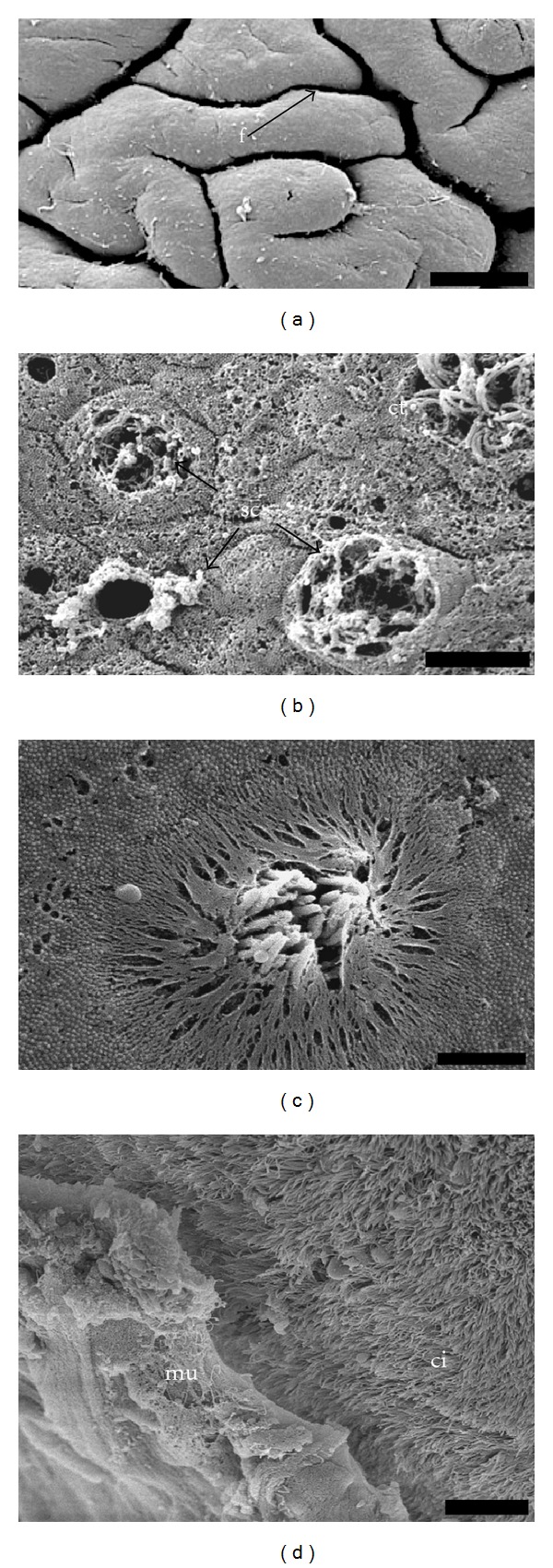
Scanning electron micrograph. (a) General view of the side foot showing the folds (f) in the external surface. Bar: 200 *μ*m. (b) Detail of the external surface of the side epithelium, ciliary tuft (ct), and opening of the secretory cells (sc). Bar: 5 *μ*m. (c) Ciliary tuft in the side foot. Bar: 2 *μ*m. (d) General view of the sole foot showing the long field of cilia (ci) and the mucus layer (mu). Bar: 10 *μ*m.

The external surface of the side foot is relatively rough containing many vertical folds that form crests and grooves (Figure 2(a)). At SEM, microvillus epithelial cells are observed alternating with the openings of secretory cells (Figure 2(b)). Moreover, cells with small ciliary tufts occur in a very low density separated from each other more than 100 *μ*m. The diameter of ciliary tufts is around 4 *μ*m, and they are comprised of approximately 30 cilia surrounded by microvilli (Figures 2(b) and 2(c)). However, the external surface of the sole foot bears a dense field of long cilia, which are generally covered by a thick layer of mucus (Figure 2(d)). This layer of mucus is important to protect the foot during locomotion as well as to help with the adhesion to the rock's surface.

### 3.2. Light Microscopy

The results of histochemistry analysis reveal the presence of different types of glycoconjugates in side and sole epithelial secretory cells and in subepithelial glands of sole foot.

#### 3.2.1. Conventional Histochemistry

**Figure 3:**
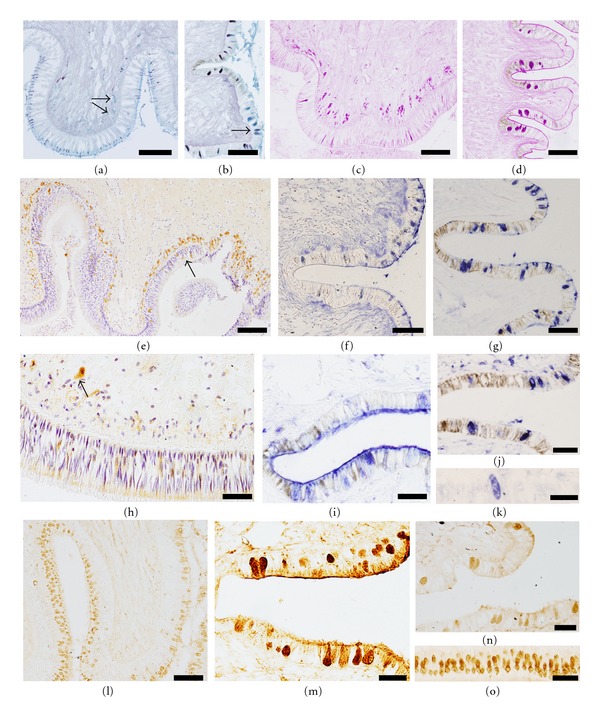
Histochemistry of the sole and side foot (sections are oriented to the bottom for the sole foot and to the right for the side foot, according their natural location) of *Haliotis tuberculata*. (a) and (b) HID/AB. The sole (a) and side (b) secretory cells in dark containing sulphated acidic glycoconjugates, moreover some subepithelial glands (a, arrows) and side secretory cells (b) stain in blue revealing the presence of carboxylated acidic glycoconjugates. Both glycoconjugates are present in a few secretory cells (b, arrow). Bar: 200 *μ*m (a), 100 *μ*m (b) (c) and (d). PAS. Presence of neutral glycoconjugates in the sole secretory cells and subepithelial gland (c), and the side secretory cells (d) Bar: 200 *μ*m (c), 100 *μ*m (d) (e) UEA I (fucose). The subepithelial glands are strongly reactive to the lectin, and a few secretory cells (arrow) are also positive. Biotinylated lectin, counterstained with hematoxylin. Bar: 100 *μ*m. (f) and (g) AAA (fucose). Some side secretory cells show positivity (f) which increases after desulphation (g) Lectin labeled with digoxigenin. Bar: 100 *μ*m. H. Con A (mannose). The apical portion of the sole epithelial cells and subepithelial gland exhibit reactivity to mannose. Biotinylated lectin, counterstained with hematoxylin. Bar: 50 *μ*m. (i) GNA (mannose). After desulphation GNA binds to some side secretory cells. Lectin labeled with digoxigenin. Bar: 50 *μ*m. (j), and (k) PNA (Galactose beta 1-3 N-acetyl-galactosamine). After desulphation the number of side secretory cells increases (j) and a few sole secretory cells appear (k) Lectin labeled with digoxigenin. Bar: 50 *μ*m. (l) and (m) WGA (N-acetyl-glucosamine). The weak reaction changes after desulphation, increasing intensity and number of sole (l) and side (m) secretory cells. Subepithelial glands appear weakly stained (l). Biotinylated lectin. Bar: 100 *μ*m (l), 50 *μ*m (m) (n), and (o) DBA (N-acetyl-galactosamine). The number of positive side secretory cells increases after desulphation (n) and a high number sole secretory cells appear (o) Bar: 50 *μ*m.

Along the foot epithelium quite abundant epithelial secretory cells are stained with alcian blue (AB). However, subepithelial glands show a weak positivity. After desulphation AB positivity disappears, indicating that most of the AB-positive epithelial secretory cells contain mostly glycoconjugates with O-sulphated groups. The high iron diamine/alcian blue technique also supports this result (Figures 3(a) and 3(b)). In addition, some subepithelial glands (Figure 3(a)) and side secretory cells (Figure 3(b)) are stained in blue with this technique, which indicates that they contain carboxylated glycoconjugates. Moreover, at the border between the side and the sole foot, two types of acidic glycoconjugates are detected in the same secretory cell (Figure 3(b)). With the periodic-acid-Schiff (PAS) technique, a strong reaction is found in the subepithelial glands (Figure 3(c)). The number of positive epithelial secretory cells varies from scarce in the sole foot (Figure 3(c)) to moderate in the side foot (Figure 3(d)). As sections treated with alpha amylase-PAS technique exclude the presence of glycogen in these cells, the PAS positivity is due to neutral sugars and/or sialic acids.

#### 3.2.2. Lectin Histochemistry

Results have been organized by grouping lectins with similar carbohydrate specificity. Regarding L-fucose binding lectins, L-fucose-residues are detected in the subepithelial glands and the sole secretory cells (Figure 3(e)) with the three lectins assayed (AAA, LTA, and UEA-I). In the side foot, only the lectin AAA binds moderately to some epithelial secretory cells (Figure 3(f)), but increasing the number of positive cells and the intensity of the staining after desulphation treatment (Figure 3(g)). In the case of mannose-binding lectins (ConA and GNA), strong positive labeling with both lectins is found in the apical edge of all sole epithelial cells. Moreover, these two lectins bind to the subepithelial glands (Figure 3(h)). In contrast, sole secretory cells are unreactive with both of them even after desulphation, whereas the side secretory cells are reactive with GNA only after such treatment (Figure 3(i)). With the lectin PNA no binding is detected in the sole foot whereas scarce side secretory cells are positive. After desulphation the number of positive side secretory cells increases, and a few sole secretory cells are also positive (Figures 3(j) and 3(k)). The N-acetyl-glucosamine binding lectin (WGA) binds weakly to some secretory cells. However, the staining pattern changes completely with the desulphation treatment, which displays a strong reactivity for this lectin in the sole and side secretory cells (Figures 3(l) and 3(m)). Moreover, a small number of cells is also weakly stained in the subepithelial glands following this treatment (Figure 3(l)). Concerning the lectin DBA that recognizes the terminal N-acetyl-galactosamine, no labeling is found in either the subepithelial glands or the sole secretory cells. However, some secretory cells of the side foot are reactive with DBA, and the number of positive cells increases after desulphation (Figure 3(n)). With this treatment, a high number of positive epithelial secretory cells is found in the sole foot (Figure 3(o)) whereas the subepithelial glands remain unreactive.

Lectins specific for sialic acid (SNA and MAA) do not bind to any type of cell in the foot epithelium of *Haliotis tuberculata*, suggesting that sialic acid is not present in this tissue. In this case three different concentrations (10, 25, and 50 *μ*g/mL) were assayed to verify the results. However, staining with these lectins is detected in the connective tissue, which confirms that the technique has been properly performed.

### 3.3. Transmission Electron Microscopy

Under the electron microscopy, different types of epithelial and secretory cells are found between the side and the sole foot. The variability of those cell types indicates differences at the functional level.

#### 3.3.1. Epithelial Cell Types

**Figure 4:**
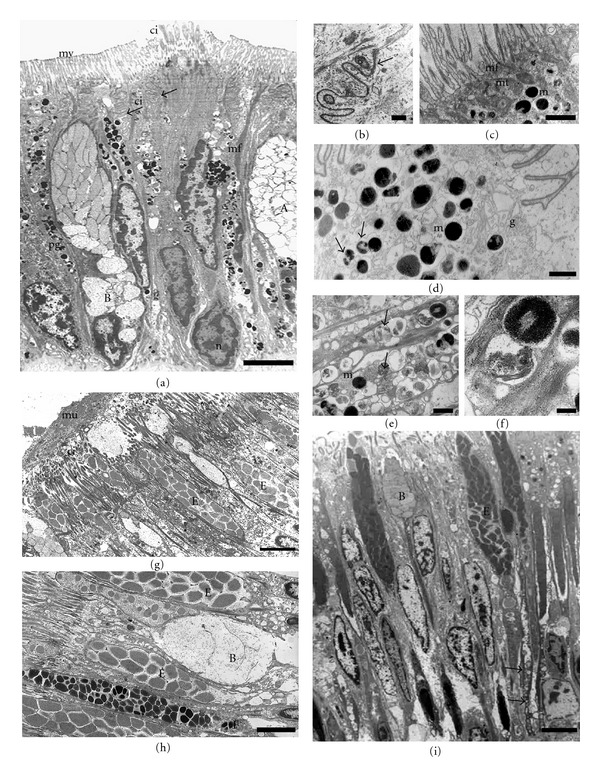
Transmission electron microphotographs of the side (a–f) and sole (g–i) foot epithelia. (a) General view showing secretory cells (types (a) and (b)) and pigmented epithelial cells with a prominent microvillus border (mv), a small group of cilia (ci) projecting from the apical surface. Note the highly infolded lateral membrane (arrows). Nucleus (n), pigmented granules (pg). Bar: 5 *μ*m. (b) Detail of the apical region of adjacent cells, cell junctions which a zonula adherens can be observed (arrow). Bar: 0.5 *μ*m. (c) Detail of microvillus border, microfilaments (mf) extend from the apical surface and comprise the core of the microvilli. At the apical surface mitochondria (mt) and melanosomes (m) are concentrated. Bar: 5 *μ*m. (d) Melanosomes (m) and partially melanized granules (arrows) from a pigmented cell located on a groove. Golgi complex (g) Bar: 1 *μ*m. (e) A pigmented epithelial cell located on the crests showing phycobilin-like pigmented granules (arrows) and a few melanosomes at different developmental stages (m) Bar: 0.3 *μ*m. (f) Detail of both pigmented granules. Bar: 0.1 *μ*m. (g) The mucus (mu) layer over the ciliated sole cells. Cilia (ci) Bar: 3 *μ*m. (h) Three types of secretory cells (B, E, F) interspersed with ciliated cells. Bar: 2 *μ*m. (i) General view of the ciliated sole foot epithelium. Type (b) and (e) secretory cells are located among ciliated epithelial cells. Observe the long necks (arrows) extending to the basal membrane. Bar: 3 *μ*m.

The side epithelial cells are typically columnar with the lateral membrane highly infolded (Figures 4(a) and 4(b)). Adjacent cells are joined together in their apical regions by cellular junctions with the appearance of zonula adherens; moreover, a high degree of interdigitation occurs beneath the junctional complex (Figure 4(b)). Small ciliary tufts, probably originated from a single cell, are sparsely distributed among the microvilli (Figure 4(a)). Bundles of microfilaments criss-cross the cytoplasm of the cell and make up the core of microvilli (Figure 4(c)). In addition, Golgi complex and mitochondria are mainly distributed in the apical part of the cytoplasm (Figures 1(a), 4(c), and 4(d)). The nuclei are located either in the centre or at the base of the epithelial cells. Small clumps of electron-dense chromatin are distributed throughout the nucleoplasm, particularly associated with the inner nuclear membrane (Figure 4(a)). Moreover, it has distinguished two different side epithelial cells which contain two types of pigments. On the grooves, deeply pigmented melanin cells containing a large number of cytoplasmic melanosomes are found (Figures 4(a), 4(c), and 4(d)), which cause the brown color of these areas observed at light microscopy or even macroscopically. In addition to rounded melanosomes, there are many partially melanized organelles that contain two different electron-dense components and may correspond to an early stage of melanosome development (Figure 4(d)). Pigmented melanin cells also show many microfilaments as well as an extensive Golgi complex with numerous vesicles intermingled with the melanosomes (Figure 4(d)). On the crests, the most common feature of the epithelial cells is the varied granular content, from electron dense to completely electron lucent. These cells have a reduced content of melanosomes and most of them are partially melanized (Figure 4(e)). Due to their location on the crests, the other vesicles containing granular or finer material may correspond to those containing a phycobilin-like pigment (Figure 4(e)), as previously observed by fluorescence microscopy [29].

The sole epithelial cells show differences in morphology and pigmentation relative to the side foot epithelium. The most noteworthy features of the sole epithelial cells are the lack of pigmented cells and the profusion of cilia on their apical domain (Figures 4(g) and 4(h)). A mucus layer forming a blanket over the top of the ciliated cells can also be observed (Figure 4(g)).

#### 3.3.2. Secretory Cell Types

**Figure 5:**
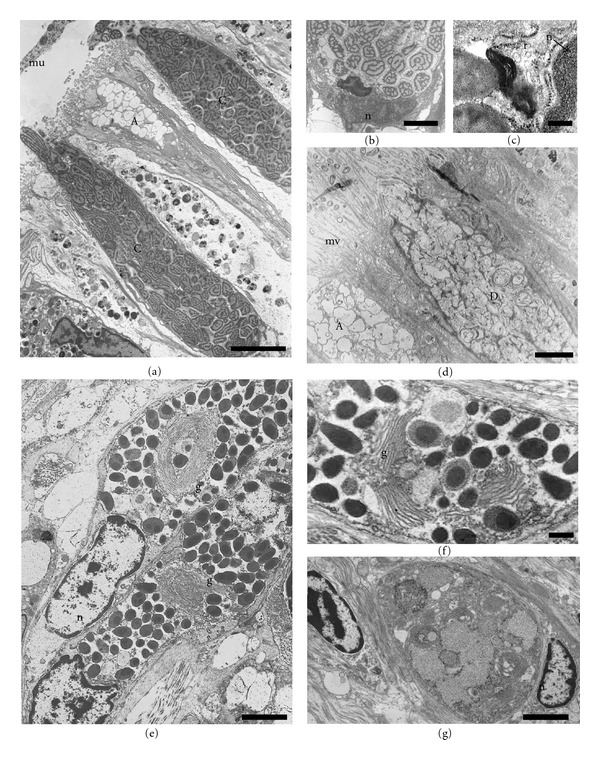
Transmission electron microphotographs of different types of side secretory cells (a–d) and subepithelial glands (e–g). (a) The characteristic secretory granules of the type C remain outside the cell, forming the mucus (mu). Type A with partially secreted granules can be observed. Bar: 5 *μ*m. (b) Basal region of the type C cell showing a body with tightly packed membranes below the nucleus (n) Bar: 2 *μ*m. (c) Detail of latter microphotograph showing a prominent rough endoplasmic reticulum (r) between nucleus (n) and the body. Bar: 0.2 *μ*m. (d) A Type D cell with swirled membranous vesicles and a type A cell. Bar: 3 *μ*m. (e) Subepithelial multicellular gland with a well-developed Golgi complex (g) and dense secretory granules associated. Bar: 2 *μ*m. F. Detail of the Golgi complex (g) in the subepithelial multicellular gland. Bar. 0.5 *μ*m. (g) Subepithelial unicellular gland. Bar: 2 *μ*m.

Along the side foot, epithelial secretory cells are scattered among epithelial cells. They are similar in shape and appearance to goblet cells, and characterized by an apical surface swollen with secretory granules and a narrow basal region with the nucleus (Figure 1(a)). By TEM, four different types of secretory cells (A, B, C, and D) are found, which are mostly distinguished by the appearance and electron density of secretory granules. The secretory granules of the cells identify here as type “A” are completely electron-lucent, very tightly packed and occupy the entire cytoplasm (Figures 4(a), 5(a), and 5(d)). The type “B” secretory cell has electron-lucent granules with finely granular material and a small nucleus located basolaterally (Figure 4(a)). The type “C” contains secretory granules with an unequal distribution of electron-dense and electron-lucent material (Figure 5(a)). Their nuclei are small and appear compressed in the basal part of the cell, where bodies with tightly packed membrane can be observed (Figure 5(b)). Moreover, a prominent rough endoplasmic reticulum is found close to the nucleus (Figure 5(c)). Extrusion of secretory material from the cell is often observed, and, occasionally, a number of granules remain apparent outside (Figure 5(a)). The cell type “D” possesses atypical secretory granules consisting of highly abundant tightly packed and swirled membranes, which may correspond to residual material (Figure 5(d)).

Three types of secretory cells are also observed in the sole foot epithelium. One of them is similar to the type B described in the side foot epithelium (Figure 4(h)). The second one is more abundant and presents a secretory product similar to that of type B cell, but it is concentrated in denser granules. We identify this new secretory cell as type E (Figures 4(g) and 4(h)). The third type (type F) possesses small and dense grains (Figure 4(h)), with higher electron density in the center than in the periphery.

In addition to these three types of epithelial secretory cells, clusters of secretory cells localized in a subepithelial position form multicellular glands on the sole foot (Figure 5(e)). Under TEM microscopy, these multicellular glands contain very dense granular material, which could be discharged on the sole via neck openings located between epithelial cells (Figures 1(b) and 4(i)). These secretory cells are characterized by a very well-developed Golgi complex arranged in a circular manner (Figures 5(e) and 5(f)). Occasionally, subepithelial unicellular secretory glands have been found with granules that resemble those found in the epithelial secretory type B cells (Figure 5(g)).

## 4. Discussion

### 4.1. Epithelial Cells

The foot epithelium of *Haliotis tuberculata* presents unique features in both the side and the sole foot epithelia. The side epithelial cells are highly pigmented with melanin and phycobilin granules and possess a prominent microvillus border. A similar epithelium has been described in the side foot of other gastropod, *Patella vulgata* [14], but, in this case, only melanin granules were observed. Pigmented cells containing mature and nonmature melanosomes, similar to those observed in the epithelium of the abalone (present results), have been reported in *Sepia officinalis* [34]. However, in the case of similar pigmented cells which provided a red-purple color to the skin of *Aplysia californica* [35], no melanin granules were described. Moreover a high abundance of bluish-green pigment was observed in the side epithelial cells located on the crests of the folds, which has the characteristics of the phycobiliprotein previously detected by using a fluorescence microscopy [29]. This type of pigmented granules content electron-lucent finely granular material, that has not been described before in any other gastropod. In a review on the biochromy of the Mollusca, Fox [36] described in *Haliotis* a bilichrome pigment known as haliotisrubin, which is accumulated from the consumption of red algae. Therefore, a dietary origin is also plausible for the pigment we detected in the epithelium of *Haliotis tuberculata*.

Moreover, the side foot of *Haliotis tuberculata* has been also characterized by the presence of toxins in a type of secretory cell [29]. It could be possible that pigmentation together with toxicity play an important ecological role as defense against depredators.

The presence of a brush border in the side epithelial cells is, in general, indicative of absorptive functions, even when the cell has another function. It has been demonstrated that the molluscan integument can act as a site for the active exchange of ions and metabolites [2]. Endocytosis processes have also been described in the foot epithelium of a terrestrial gastropod [37] and in the foot of the limpet [38]. Nevertheless, despite presenting prominent microvilli, no evidence for endocytosis through the apical edge in *Haliotis tuberculata* epithelial cells has been found in this study. However the presence of mitochondria mainly distributed at the epithelial cell's surface could suggest high metabolic activity.

The sole foot epithelium of *Haliotis tuberculata* (present results) and of other aquatic gastropods [see [28] for review] is characterized by the abundance of ciliated cells that are probable used to distribute the mucus for mucus gliding locomotion [21]. Some similar scattered cells have been found in the side foot, but the significance of their function is not well understood in this part of the body. In *Haliotis*, sensory and water flux recirculation functions were described for the ciliated cells of the tentacles [6, 28]. Other feature of the sole foot is the presence of a more prominent mucus layer than in the side foot, which is also important in locomotion and adhesion to the substrate.

### 4.2. Epithelial Secretory Cells and Subepithelial Glands

Histochemical studies have revealed the presence of epithelial secretory and subepithelial glandular cells in the gastropod foot, but their number and chemical composition varied greatly among the different species studied [28]. This is probably due to the considerable variety of habitats they occupy and to their different modes of life. It has been proposed that limpets can secrete different forms of mucus for mobility or adhesion to rocks [23] in response to their tidal activity cycles. However, we cannot expect the same for *Haliotis* who lives in the infralittoral area never exposed to tide cycles [39].

Concerning the foot of *Haliotis tuberculata*, present results corroborate the occurrence of neutral and acidic (mostly sulphated) glycoconjugates that we have previously described [29]; in addition, in this work the presence and distribution of sugar residues in the oligosaccharide side chains of glycoconjugates in its pedal epithelium were investigated by using specific lectins.

Following classical carbohydrate histochemical techniques, most epithelial secretory cells along the foot of *Haliotis* are rich in acidic sulphated glycoconjugates (present results), which is in neatly agreement with previous studies describing that sulphate is a major component of the gastropod mucus [14, 15, 18, 40]. By other hand, the subepithelial glands showed intense staining with PAS and weak affinity for AB, indicating that these cells produce a mixture of glycoconjugates with the neutral glycoproteins predominating over acidic glycoconjugates. In contrast in the foot epithelium of some nudibranch, the subepithelial glands called long-necked cells were the origin of the acidic glycoconjugates [17] whereas the epithelial secretory cells were PAS positive at both light and ultraestructural [41] levels. The different histochemical pattern of these studies may have been caused by the variety of secretory cell types distributed over the gastropod foot.

In *Haliotis*, the subepithelial glands, together with the sole secretory cells, showed reactivity with the three fucose-specific lectins assayed; moreover, AAA binding was also found in the side secretory cells. The lectins UEA I and LTA label preferentially fucose residues located in the outer region of the oligosaccharide chains [42], whereas AAA can also bind to those fucose residues alpha(1–6) linked to the innermost N-acetyl-glucosamine of the core of N-linked oligosaccharides [43]. Therefore, our findings suggest that the fucose residues present in the side foot and recognized by AAA are linked to the core region of N-linked oligosaccharides. This kind of fucosylation has been described in N-glycoproteins from different aquatic and terrestrial species of gastropods [44].

Mannose-specific lectins (ConA and GNA) bind to the apical portion of the sole ciliated epithelial cells as well as to the subepithelial glands. A moderate binding of these lectins to the apical portion of ciliated duct cells has been described in the digestive tubules of *Mytilus* [26]. The lectin ConA binds to some specific classes of N-glycans and is not known to bind O-glycans on animal cells glycoproteins [45]. Our results suggest that the subepithelial glands contain mainly N-glycoproteins. In the side secretory cells mannose residues are only detected with GNA after desulphation treatment demonstrating that sulphated mannose is present in a terminal position.

Labeling with the lectins WGA and DBA indicates the presence of sulphated residues of N-acetyl-glucosamine and N-acetyl-galactosamine, respectively, in both the sole and side secretory cells, but not in the subepithelial glands. These monosaccharides are fundamental constituents of sulphated glycosaminoglycans such as heparan sulphate, heparin, and chondroitin sulphate, and of sulphated mucins. Sulphated glycosaminoglycans and mucins have been described in other gastropods [46–48] and could be responsible for an increased viscosity of the secretions [21, 48]. The occurrence of acidic sulphated glycoconjugates in the epithelial secretory cells, but not in the subepithelial glands, found with lectins, agrees with our results with classical histochemistry.

Glycans terminating with the sequence galactose (beta1-3) N-acetylgalactosamine (PNA reactivity) were found in the side secretory cells only after desulphation treatment. A similar result was described by Robledo et al. [26] in the digestive gland of *Mytilus galloprovincialis*, which indicates the presence of terminal sulphated galactose in O-linked oligosaccharides [49], most likely in sulphated mucins. If the mucus secreted by the side foot has some similar characteristics that one secreted by the digestive gland that means that it could be indicated that it is involved in protective and lubrication functions.

Our results with the lectins SNA and MAA demonstrate that sialic acid is not present in both side and sole foot epithelium and in the subepithelial glands of *Haliotis tuberculata*; however, it was detected in the connective tissue of this gastropod. It has been long claimed that the sialic acid is not present in gastropods, being replaced with N-acetylmuramic acid [5, 50]; however, it was later detected in different mollusks by using biochemical [51, 52] and histochemical (present results) methods.

In general, our data suggest that the sole subepithelial glands contain mainly N-glycoproteins. In contrast, the sole secretory cells are characterized by the presence of sulphated glycosaminoglycans which could be constituents of proteoglycans, and the side secretory cells are rich in mucins, mostly sulphated. The sole mucus is a mix of N-glicoproteins and proteoglycans that *Haliotis* probably uses to increase the protection and adhesion to the sustrate. However the side mucus rich in sulphated mucins typically increase the viscosity of the mucus and are very important in protection.

In order to better understand the differences observed in the glycoconjugates composition among the secretory cell types of *Haliotis tuberculata* foot epithelium, we addressed an ultrastructural study. The obtained results allowed us to identify seven types of secretory intraepithelial cells and two types of subepithelial glands, with the latter only present in the sole foot. The secretory cells contain vesicles that are quite variable in appearance and electron density. The structure of these cells is similar to that previously described for other secretory cells [6, 53], with the exception of the types C and D, which are characteristic of the side foot. Secretion granules with a similar ultrastructure to those present in type C, and D cells were not previously reported.

In conclusion, the variations between the side and sole *Haliotis tuberculata* foot epithelia concerning the ultrastructure of the epithelial and secretory cells, together with the different types of glycoconjugates found on both parts, indicate possible functional differences between both areas of the abalone integument.
